# Spontaneous splenic rupture: case report and review of literature

**DOI:** 10.11604/pamj.2020.37.36.25635

**Published:** 2020-09-08

**Authors:** Tariq Ahbala, Khalid Rabbani, Abdelouahed Louzi, Benasser Finech

**Affiliations:** 1 General Surgery, Mohammed VI University Hospital Center of Marrakech, Marrakech, Morocco

**Keywords:** Splenic rupture, atraumatic, unknown etiology

## Abstract

Splenic rupture is a potentially life-threatening condition, often associated with chest or abdominal trauma. Spontaneous rupture is very rare and is usually reported as being secondary to underlying pathological conditions. We report a case of atraumatic splenic rupture in a patient with no underlying disease pathology. This case should remind the emergency physician spontaneous splenic rupture should be considered in the differential diagnosis of unexplained acute abdominal pain.

## Introduction

Splenic rupture is mainly caused by trauma. But in some rare cases, it can also occur without obvious trauma, known as atraumatic splenic rupture (ASR) or spontaneous spleen rupture. ASR is often life threatening due to the delay of diagnosis and treatment.

## Patient and observation

**Figure 1:**
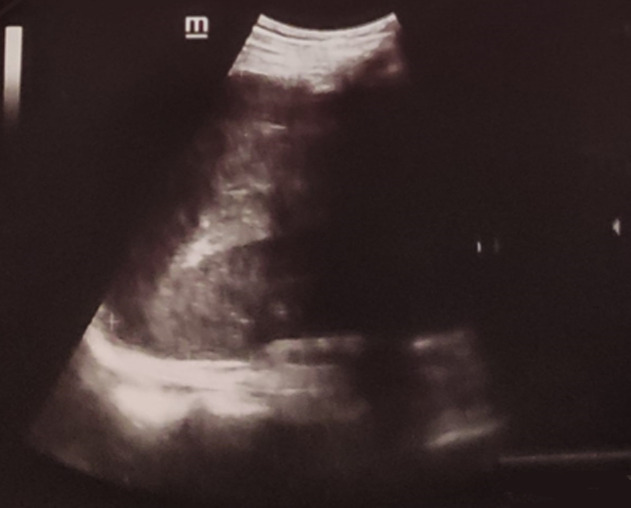
ultrasound showing a hypoechogenic range of the splenic compartment

**Figure 2:**
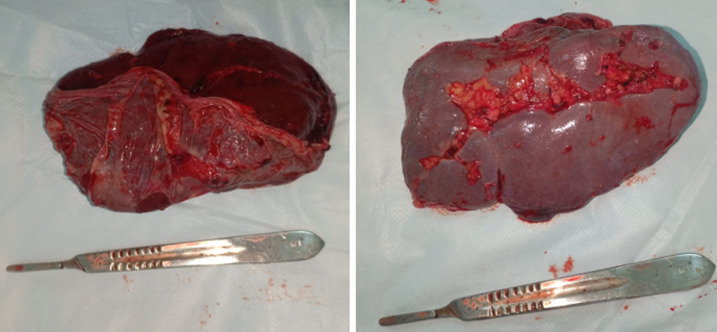
macroscopically healthy spleen

A 62-year-old man, with chronic smoking, arrived at the emergency department complaining of abdominal pain with sudden-onset. On arrival, there was a collapse, blood pressure 90/60mmHg and pulse 109/min, the patient was pale, apyretic and had an abdominal defense. The hemoglobin was at 8 g/dl and WBC at 19000/mm^3^. After resuscitation measures and transfusion of 4 units of red blood cells transfusions, the ultrasound examination showed echogenic peritoneal effusion with hypoechognic mass of the left hypochondrium (Figure 1). As the patient remained haemodynamically unstable, he underwent an exploratory laparotomy. During laparotomy, there was a hemoperitoneum related to complete decapsulation of spleen (Figure 2). The decision was made to proceed to a splenectomy. Histological examination confirmed the non-pathological aspect of a decapsulated spleen. The patient’s hospital course was unremarkable. The patient received pneumococcal, meningococcal and haemophilus vaccinations and was discharged on life-long penicillin prophylaxis.

## Discussion

Atraumatic splenic rupture was first documented in the 19^th^ century. The first cases of spontaneous splenic rupture were described by Laseter *et al*. [1] in 2004 and Badenoch *et al*. [2] in 1985. The real cause of the rupture has not yet been clearly identified [3]. The incidence rate of ASR has not been clarified. Liu J *et al*. showed that the incidence of ASR was 3.2% (8/251) [4]. ASR are twice as common in men. The age varies from 2 to 81 years (average = 42 years). In about a third of cases, there are signs of shock at the first examination. In 8% of cases, patients die before being operated on and the diagnosis is only made at autopsy [5]. Three mechanisms were involved in the process: the increase in intrasplenic tension linked to cell hyperplasia and engorgement; compression by the abdominal muscles during sneezing, coughing or defecating; vascular occlusion by hyperplasia of the endothelial reticulum responsible for infarction associated or not with a subcapsular hematoma [6]. The etiology of atraumatic rupture of the spleen can be examined under six subgroups namely, I) infectious, II) neoplastic, III) inflammatory, IV) congenital or structural, V) iatrogenic and VI) idiopathic [7]. Spontaneous rupture of the normal spleen represents a problem in diagnosis. In the absence of trauma, diagnosis of splenic rupture is not always made by considering just the classic signs and symp-toms of left upper quadrant (LUQ) pain, guarding and haemodynamic instability [8]. The existence of abdominal pain and painful massive splenomegaly points to splenic involvement which must be confirmed urgently by ultrasound, which is the first line examination. However, computed tomography presents better sensitivity for the lesion assessment [9]. In terms of treatment, splenectomy is a radical cure for spontaneous rupture of the spleen. However, the morbidity of splenectomy, improved surgical techniques and intensive care, and the role of the spleen in the immune response have allowed us to provide conservative treatment. In some cases, this seems to be another option: hemodynamic stability, resort to blood transfusions with less than 2 red blood cell particles, daily routine and biological clinical monitoring, rest and hospitalization in departments near the surgery center treatment [10].

## Conclusion

Spontaneous splenic rupture without a history of trauma is an uncommon life-threatening abdominal emergency. The pathogenesis of the disease remains unclear. In patients with atraumatic left hypochondrial pain and low hemoglobin, splenic rupture should be kept in mind.
